# Demand for emergency health service: factors associated with inappropriate use

**DOI:** 10.1186/1472-6963-7-131

**Published:** 2007-08-18

**Authors:** Maria LV Carret, Anaclaudia G Fassa, Ichiro Kawachi

**Affiliations:** 1 Department of Public Health, Catholic University of Pelotas, Pelotas, RS, Brazil, Rua Félix da Cunha, 412, 96010-000, Pelotas, RS, Brazil; 2 Department of Social Medicine, Post-Graduate Program in Epidemiology, Federal University of Pelotas, Pelotas, RS, Brazil, Av. Duque de Caxias, 250 - 3^rd ^floor, 96030-002, Pelotas, RS, Brazil; 3 Department of Society, Human Development, and Health, Harvard School of Public Health, 677 Huntington Avenue, Kresge Building 7^th ^Floor, Boston, MA 02115, USA

## Abstract

**Background:**

The inappropriate use of emergency room (ER) service by patients with non-urgent health problems is a worldwide problem. Inappropriate ER use makes it difficult to guarantee access for real emergency cases, decreases readiness for care, produces negative spillover effects on the quality of emergency services, and raises overall costs.

**Methods:**

We conducted a cross-sectional study in a medium-sized city in southern Brazil. The urgency of the presenting complaint was defined according to the Hospital Urgencies Appropriateness Protocol (HUAP). Multivariable Poisson regression was carried out to examine factors associated with inappropriate ER use.

**Results:**

The study interviewed 1,647 patients over a consecutive 13-day sampling period. The prevalence of inappropriate ER use was 24.2% (95% CI 22.1–26.3). Inappropriate ER use was inversely associated with age (P = 0.001), longer stay in the waiting room, longer duration of symptoms and morning shift. However, the determinants of inappropriate ER use differed according age groups (P value for interaction = 0.04). Within the younger age-group (15–49 years), inappropriate ER use was higher among females, patients who reported visiting the ER because there was no other place to go, patients reporting that the doctor at the regular place of care refused to attend to them without a prior appointment, and individuals who reported that the PHC clinic which they use is open for shorter periods during the day. Among older patients (50+ years), those with highest level of education, absence of self-reported chronic diseases and lack of social support were more likely to engage in higher inappropriate ER use.

**Conclusion:**

Efforts should be made to redirect inappropriate ER demand. Besides expanding access to, and improving the quality of primary and secondary care, it is important to mobilize social support for older patients, to enhance the relationship between different levels of care, as well as to develop campaigns to educate the public about the appropriate use of medical services.

## Background

The inappropriate use of emergency room (ER) service by patients with non-urgent health problems is a worldwide problem, both in countries with publicly funded health systems as well as in those with private security systems [1-7].

The inappropriate use of theses services makes it difficult to guarantee access for real emergency cases, decreases the readiness for care, produces negative spillover effects on the quality of emergency services, and raises overall costs [6-9]. However, rationalizing the demand for ER services also depends on the appropriate use of services at other health system levels, e.g. improving access to primary health care (PHC) for preventive services, periodic consultations, and referral to specialists or hospital services when needed [10,11]. Primary health care is the appropriate setting for continuous care [12]. Continuity of care, in turn, improves the doctor-patient relationship, increases treatment adherence and follow-up, facilitates health education and decreases the inappropriate use of emergency services, hospitalization rates and the number of tests requested [13,14].

The prevalence of inappropriate demand for emergency health services depends on the criteria used to define appropriateness [1,14,15]. According to the literature, patients who inappropriately seek emergency services are mainly young [7,16,17], predominantly women [16,18], and are not referred to the service by a health professional [16].

Several factors may lead patients to choose emergency services instead of primary and specialized health services [19,20], including: the desire to receive care on the same day [6,19], the possibility of being attended to in a setting where it is possible to do laboratory and other tests [3], and the belief that ER services are able to solve complex types of health problems [15,21]. However, patients frequently underestimate the importance of continuous care, and they often lack the knowledge that their decision to seek ER services may result in the excessive use of medicines and diagnostic tests [9,13].

In Brazil, the national health care system is characterized by the universality of care (free access), a hierarchical structure with three levels of increasing complexity (primary, secondary and tertiary levels), and an integrated approach to delivering care for all types of health needs [22].

Pelotas is a medium size city located in Southern Brazil, with 323,158 inhabitants, 93% of whom live in urban areas and where the prevalence of illiteracy is 6%. The city has a public health system including 50 primary health care centers (PHC) spread across the city, each staffed (at minimum) by a general physician, a nurse and a receptionist. The secondary level of care comprises specialist physicians, who work in ambulatory clinics, while the tertiary level of care comprises four hospitals and one ER [22].

The objective of this study was to identify the prevalence and risk factors for inappropriate use of ER service in the municipality of Pelotas. Although inappropriate demand for ER services is a well-known problem, there are few studies addressing its causes. Thus, this study may provide important information for addressing the problem and improving health systems performance.

## Methods

We carried out a cross-sectional study of the ER service utilization in the city of Pelotas, Brazil. Data collection was conducted during the spring of 2004 to monitor ER demand 24 hours a day for 13 consecutive days (9 weekdays, 3 weekend days and 1 holiday). The sampling duration was based on priori sample size calculations (described below). All patients aged 15 years or older were included in our study. The age group was chosen because it corresponds to the age-range within which our outcomes criteria (defining inappropriate ER use) were validated. Individuals were excluded if they had communication difficulties not related to their presenting complaint, or if they were brought to the ER by the police for forensic medical exams. Individuals who returned more than once answered the questionnaire only once. Those who refused to answer the questionnaire after at least three attempts were classified as refusals.

The sample size calculations for our study assumed a prevalence estimate of 27% obtained from Oterino et al [14] which applied the same criteria for inappropriate ER use as in our study. The unexposed/exposed ratio of the independent variables varied from 1:1 to 1:5. These estimated ratios were based on Bianco et al [16] who described a wide range of variables related to access and utilization of services. The sample size was estimated to detect relative risks higher than 1.5 with a confidence level of 95% and statistical power of 80% for all the associations examined in our study. The largest sample size required was for examining the association between source of referral to the ER and inappropriate ER use, which was estimated to require 1,158 subjects (assuming an unexposed/exposed ratio of 1:5, where self-referrals were considered as the 'exposed' category). We inflated our power size estimates by 10% to allow for refusals, as well as by a further 15% to allow for adjustment by confounding factors. Our final sample size estimate of 1,465 subjects allowed for prevalence estimates with a 3 percent margin of error.

The urgency of the presenting complaint was defined according to the Hospital Urgency Appropriateness Protocol (HUAP), a previously-developed standardized and validated set of criteria [23]. This definition does not take into account the relevance of the clinical care provided at the emergency service, assuming that appropriate use occurs when the presenting complaint was deemed urgent. According to HUAP criteria, a visit was deemed to be urgent if it fulfilled at least one of the following criteria:

1. Criteria of severity

1.1. Patients with one of the following conditions (sudden or very recent onset): (a) loss of consciousness; (b) disorientation; (c) coma; (d) sensory loss; (e) sudden loss of sight or hearing.

1.2. Patients with one of the following conditions: (a) pulse rate alteration – <50 or >140 bpm; (b) arrhythmia; (c) blood pressure alteration; (d) electrolyte or blood gas alterations (not including patients with chronic alterations of these parameters, such as: chronic kidney failure, chronic respiratory disease, etc); (e) persistent fever – 5 days or more, not controlled after treatment in primary care; (f) active hemorrhage; (g) sudden loss of functional capacity of any part of the body;

2. Criteria for treatment

One of the following procedures: (a) intravenous drugs administration (except to maintain IV access); (b) oxygen administration; (c) setting with plaster casts – except for bandaging; (d) surgical intervention or procedure.

3. Criteria for diagnostic intensity

One of the following: (a) monitoring of vital signs every 2 hours; (b) radiology of any type; (c) laboratory tests – except blood sugar in diabetic patients seeking care for reasons other than diabetes and glycemia tests with glucose test sticks; (d) electrocardiography – except in patients with chronic cardiac disease who presented for unrelated problems.

4. Other criteria

One of the following: (a) patient has been under observation in the ER for twelve hours or more; (b) patient is admitted to hospital or transferred to another hospital or dies in ER;

5. Criteria used only for patients who self-referred

One of the following: (a) has had an accident (traffic, work-related, in public place,...) and needs to be examined; (b) symptoms suggesting vital emergency: e.g. chest pain, dyspnea with rapid onset, acute abdominal pain; (c) patients with a known condition which usually leads to hospitalization; (d) the patient's physician advised that he/she needed to go to the emergency service if symptoms appear; (e) patients who required quick medical attention, and the hospital was the closest center; (f) other circumstances in self-referred patients – specify.

We collected demographic data – age, sex, skin color and marital status (living with or without a partner). Socioeconomic variables included level of education (years of formal education) and ownership of material assets based on quintiles of an index derived from principal components analyses. Some variables indicating health need were also evaluated: self-reported chronic diseases, self-reported depression and self-reported health status.

Variables relating to PHC utilization and access included: consultation with a primary care provider within the past year; presence of a regular doctor (i.e. the patient went most times to the same doctor, knew his/her name and visited him/her at least once in the previous 12 months); defined place for consultation (the patient had a defined place go, and visited it at least once in the previous 12 months); health insurance status; the reason for choosing the ER service was that he/she was unable to make an appointment elsewhere; number of shifts per day at the PHC service; whether or not the doctor at the regular place of care refuses to attend patients; access to prescription drugs at the primary care clinic (received any drug prescribed by the PHC provider); had to wait more than 30 days to have a consultation with the specialist, or to receive diagnostic tests in primary care, degree of satisfaction with the health system [24], previous medical evaluation for the same reason prompting the subject to visit the ER, and whether or not the subject was referred by a health worker.

Several variables pertaining to the ER visit were also collected: time elapsed from the onset of symptoms to arrival in the ER (symptom duration); time in the waiting room (from arrival in the ER to the consultation); and the shift during which the ER visit took place. Others variables collected included: social support (evaluated by the availability of someone, e.g. a relative or a friend, to schedule and accompany the patient during the visit), self-reported urgency (whether the problem required immediate medical attention, and was life-threatening or not), and number of consultations in the emergency service in the last three months.

The fieldwork was carried out by 12 trained interviewers (4 during each shift), who were not briefed about the objectives of the study. The field work was supervised by the lead author. Most of the interviews were conducted in the emergency service, but if there was an impediment, the interviews could also be conducted at the hospital or at the patients' homes. In each shift one research assistant filled the patients' identification data, capturing all admitted patients. The variables were coded by the interviewers, and the research coordinator reviewed the work. Epi-Info software was used to double enter the data. Analyses were carried out using Stata 8.0.

The descriptive analysis included calculation of prevalence, means, and standard deviations (SD) of all variables. Crude associations were evaluated by the Chi-square, test for heterogeneity or linear trend. Multivariate analysis was carried out by Poisson regression, using robust variance estimates as appropriate for binary outcomes with high prevalence [25]. The analysis followed a hierarchical modeling strategy, including demographic variables in the first step, socioeconomics variables in the second stage, self reported health needs in the third stage; and, in the final stage, variables related to primary health services. Confounding factors were kept in the multivariate model when associated with the outcome at a significance level lower than 20%. All tests were two-tailed.

The project was approved by the Medical School Ethics Committee of the Federal University of Pelotas (Cocep Number: 40601115), and informed consent was obtained from all subjects. Confidentiality was ensured. This Ethics Committee is in compliance with the Helsinki Declaration.

## Results

During the period of the study, there were 1,974 visits, with 144 individuals returning more then once. Among the 1,830 patients, 71 (3.9%) were not eligible and 112 of the eligible subjects (6.4%) refused to take part in the study, resulting in a final sample of 1,647 patients. For medical or logistical reasons 112 (6.8%) completed the questionnaire outside the ER (at home or in the hospital). The overall prevalence of inappropriate use of ER was 24.2% (95% confidence interval (CI) 22.1 – 26.3). Inappropriate ER use was higher in the younger age group (15–49 years) compared to the older age group (50 years or older) (26.4 and 20.4%, respectively, P = 0.007).

The mean age of patients was 43.6 years (SD 19.5; range 15–100). Among the sample, 52.1% were female and approximately 3/4 was classified by the interviewers as having white skin color. The median level of education was five years. Almost 55.0% of all patients lived with a partner (Table 1).

**Table 1:** Sample description of the use of emergency services. Pelotas, Brazil, 2004

**Variables**	**N**	**(%)**
**Age in years (N = 1647)**		
15 – 19	158	9.6
20 – 34	471	28.6
35 – 49	411	25.0
50 – 64	321	19.5
65 or more	286	17.4
**Sex (N = 1647)**		
Male	789	47.9
Female	858	52.1
**Skin color (N = 1646)**		
White	1264	76.8
Non-white	382	23.2
**Marital status (N = 1647)**		
Living without a partner	748	45.4
Living with a partner	899	54.6
**Level of education – years of formal education (N = 1643)**		
0	197	12.0
1–4	399	24.3
5–8	660	40.2
9–11	326	19.8
12 or more	61	3.7
**Quintiles of an index derived from principal components analyses (N = 1642)**		
1 (poorest)	455	27.7
2	246	15.0
3	303	18.5
4	311	18.9
5 (richest)	327	19.9

One in every three individuals reported at least one lifetime episode of depression. Overall, 71.2% had a defined place of usual care and 28.1% subjects had a regular doctor but only 13.7% of these were referred to the ER by a doctor. One in every six subjects had health insurance, and 8.2% went to ER because it was the sole health service available.

With regard to primary care utilization, 21.4% of the patients had not used primary care, and only 20.3% reported that their PHC clinic remained open throughout the night (regular schedule from 8 am to 10 pm). Five per cent of patients reported that their PHC doctors usually refused to attend patients without a prior appointment. Out of the 668 subjects who reported needing drugs at the last PHC visit, only half had access to the medicines needed; and out of the 423 individuals who needed to have tests, 35.0% had to wait 30 days or more. In addition, among patients who were referred to a specialist by their primary care provider (N = 182), 28.0% had to wait more than 30 days for an appointment.

Only 40.7% of the patients went to the ER on the same day in which their symptoms started, and 12.0% waited more than 10 days to go. Around 1/4 of the subjects were referred to the ER by a health professional. Six percent of the patients had visited the ER three or more times in the previous three months.

Inappropriate use of the ER was 46.0% (P = 0.04) more frequent during the morning shift than the dawn hours, and directly associated with symptom duration and with time in the waiting room (P < 0.001) (Table 2).

**Table 2:** Characteristics of inappropriate use of emergency service. Pelotas, Brazil, 2004

**Variables**	**N**	**Prevalence (%)**	**Crude PR (95% CI)**	**P**
**Shift during which the ER visit took place**				
Morning (7 am – 1 pm)	416	27.9	1.46 (1.02 – 2.10)	0.04
Afternoon (1 pm – 7 pm)	556	24.6	1.29 (0.90 – 1.85)	0.16
Night (7 pm – 1 am)	522	22.2	1.16 (0.81 – 1.68)	0.41
Daybreak (1 am – 7 am)	152	19.1	1.00	
**Time taken from the onset of symptoms to arrival in the ER**				<0.001*
<1 day	670	14.8	1.00	
1 – 10 days	777	30.2	2.04 (1.66 – 2.52)	
11 days or more	198	32.3	2.18 (1.66 – 2.87)	
**Time in the waiting room**				<0.001*
Up to 5 minutes	663	16.4	1.00	
6 – 15 minutes	486	26.5	1.61 (1.29 – 2.03)	
16 – 30 minutes	312	31.1	1.89 (1.49 – 2.40)	
31 minutes or more	178	33.7	2.05 (1.57 – 2.68)	

PR: prevalence ratio, ER: Emergency Room, CI: confidence interval, * Test for linear trend

The prevalence of inappropriate consultations was inversely associated with age (P = 0.001). However, age was also a modifier of the association between other independent variables and inappropriate use of ER (P value for interaction = 0.04), which suggests that the determinants of inappropriate ER use may differ among younger patients (15 and 49 years) and older patients-(50 years or more). Thus, tables 3 and 4 present multivariate analyses stratified by age.

**Table 3:** Variables associated with inappropriate use of emergency services (15 – 49 years-old): crude and multivariable analysis. Pelotas, Brazil, 2004

**Variables**	**N**	**Prevalence (%)**	**Crude PR (95% CI)**	**P**	**Adjusted PR (95% CI)**	**P**
**First level**						
**Sex**				<0.001		<0.001
Male	513	20.9	1.00		1.00	
Female	526	31.8	1.52 (1.23 – 1.88)		1.52 (1.23 – 1.88)	
**Skin color**				0.09		0.11
White	793	25.1	1.00		1.00	
Non-white	246	30.5	1.21 (0.97 – 1.52)		1.20 (0.96 – 1.50)	
**Marital status**				0.15		0.12
Living without a partner	588	28.6	1.16 (0.95 – 1.42)		1.17 (0.96 – 1.44)	
Living with a partner	452	24.7	1.00		1.00	
**Second level**						
**Level of education – years of formal education**				0.13*		0.15*
0 – 4	210	21.0	1.00		1.00	
5–8	493	28.0	1.34 (0.99 – 1.80)		1.35 (1.00 – 1.81)	
9 or more	336	27.5	1.31 (0.96 – 1.80)		1.31 (0.95 – 1.80)	
**Third level**						
**Fourth level**						
**Visited PHC**				0.02^#^		0.17^#^
Last year	517	30.4	1.00		1.00	
Longer than one year	234	23.5	0.77 (0.59 – 1.01)	0.06	0.79 (0.60 – 1.03)	0.08
Never went there	283	21.6	0.71 (0.55 – 0.92)	0.01	0.82 (0.57 – 1.17)	0.27
**Regular doctor**				0.19		0.12
No	243	27.4	1.19 (0.92 – 1.54)		1.23 (0.95 – 1.60)	
Yes	797	23.1	1.00		1.00	
**The reason for choosing the emergency service was that he/she was unable to make an appointment elsewhere**				0.009		0.04
No	964	25.4	1.00		1.00	
Yes	76	38.2	1.50 (1.10 – 2.04)		1.38 (1.01 – 1.89)	
**Number of shifts per day at the PHC service**				0.004^#^		0.009^#^
One shift	116	31.9	1.64 (1.11 – 2.42)	0.01	1.63 (1.11 – 2.40)	0.01
Two shifts	548	29.9	1.54 (1.13 – 2.10)	0.006	1.53 (1.12 – 2.08)	0.007
Three shifts	201	19.4	1.00		1.00	
Did not use the PHC	125	18.6	0.96 (0.60 – 1.52)	0.85	0.99 (0.61 – 1.61)	0.97
**Doctor at the regular place of care refuses to attend without previous appointment**				0.009		0.04
No	947	25.4	1.00		1.00	
Yes	55	40.0	1.58 (1.12 – 2.22)		1.44 (1.02 – 2.02)	
**Subject was referred by a health worker**				0.03		0.05
No	866	27.6	1.44 (1.04 – 1.99)		1.40 (1.01 – 1.94)	
Yes	172	19.2	1.00		1.00	

PR: prevalence ratio, CI: confidence interval, * Test for linear trend, ^# ^Test for heterogeneity, PHC: primary health care, Variables in the third level and variables not presented in the first and fourth level had p-value > 0,2 and were excluded form the final model

**Table 4:** Variables associated with inappropriate use of emergency services (patients with 50+ years-old): crude and multivariable analysis. Pelotas, Brazil, 2004

**Variables**	**N**	**Prevalence (%)**	**Crude PR (95% CI)**	**P**	**Adjusted PR (95% CI)**	**P**
**Second level**						
**Level of education – years of formal education**				0.06*		0.06*
0 – 4	386	18.4	1.00		1.00	
5 – 8	167	23.4	1.27 (0.90 – 1.79)		1.27 (0.90 – 1.79)	
9 or more	51	27.5	1.49 (0.91 – 2.44)		1.49 (0.91 – 2.44)	
**Third level**						
Self-reported chronic diseases**				0.02		0.03
No	92	29.4	1.57 (1.09 – 2.26)		1.50 (1.03 – 2.17)	
Yes	513	18.7	1.00		1.00	
**Fourth level**						
**Social support**				0.009		0.05
No	250	25.6	1.52 (1.11 – 2.08)		1.40 (1.01 – 1.95)	
Yes	355	16.9	1.00		1.00	

PR: prevalence ratio, CI: confidence interval, * Test for linear trend, ** Self-reported chronic diseases: diabetes, arterial hypertension, bronchitis, asthma, emphysema, heard disease, rheumatism and others. Variables in the first level and variables not presented in the third and fourth level had p-value > 0,2 and were excluded from the final model

Table 3 shows the association between the main predictor variables and inappropriate use of the ER in the younger group (15 to 49 years-old). The prevalence of inappropriate use was more frequent among women – prevalence ratio (PR) = 1.52 (95%CI 1.23; 1.88). Patients who reported visiting the ER because they were unable to make an appointment elsewhere, as well as patients reporting that their regular doctor refused to attend to them without a prior appointment were around 40% more likely to use the ER inappropriately. Individuals, who reported that the PHC service which they use is open for shorter periods during the day, were also more likely to inappropriately use the emergency services. Those who were referred to the ER by health workers were 30% less likely to have inappropriate utilization.

Among older patients (50 years or more), those with highest level of education had higher rates of inappropriate use, although the p-value was of borderline significance. Inappropriate use of the ER was associated with absence of self-reported chronic diseases (P = 0.03) and lack of social support (PR = 1.40, 95%CI = 1.01, 1.95) (Table 4). None of the other studied variables were associated with inappropriate ER use.

Table 5 compares the urgency criterion used in our study with self-reported urgency. Of the 1,248 instances of appropriate ER, 350 patients did not report their situation as urgent. On the other hand, of the 397 inappropriate ER visits (as defined by our criteria), 250 patients reported that their situation was urgent. Based on the criteria we used, the sensitivity of self-reported urgency was 72%, specificity was 37%, the predictive positive value was 78% and the predictive negative value was 30%. Although the simple percentage of agreement was relatively good (60%), the kappa for agreement between the two variables (both coded as yes/no) was very poor (0.083).

**Table 5:** Self-reported urgency and urgency as defined by the Urgency Hospital Adequate Protocol (PAUH) comparison. Pelotas, Brazil, 2004

		**Urgency by definition**		
		**Yes**	**No**	
**Self-reported**	**Yes**	(a) 898	(b) 250	1148
**urgency**	**No**	(c) 350	(d) 147	497
		1248	397	1645

## Discussion

The results of our study indicate a significant prevalence of inappropriate ER use in the city of Pelotas, Brazil. Age was an effect modifier of the association between inappropriate ER use and other predictors. Among younger patients (<50 years), the prevalence of inappropriate use was higher among women, those who visited the emergency service because they were unable to make an appointment elsewhere, those who reported that their usual PHC clinic was opened for shorter hours, those who reported that their primary care providers refused to attend to patients without a prior appointment, and those who were not referred to the ER by health workers. For older patients (50+ years) the absence of chronic diseases and lack of social support were the main factors associated with inappropriate ER use. The study also showed that self-reported urgency is a poor indicator of appropriateness.

Pelotas has only one emergency service [22] that attends all public demand, as well as the majority of patients with private health insurance. There was a low percentage of missing data and refusals, and it is likely that our sample was representative of ER utilization within the city.

There are several criteria for determining the appropriateness of ER service utilization, including observation time needed, health professionals' perceptions, and resources required for medical evaluation. The HUAP is a validated and widely used set of criteria [23]. Nonetheless some level of misclassification is possible, e.g. classifying some inappropriate use as appropriate (false-positive). This misclassification would tend to bias any association towards the null. A validation study using experts as the gold standard showed that 55% of the cases were inappropriate compared with only 24% considered inappropriate by the HUAP. The study also reported that only in one case did the HUAP classify a visit as inappropriate when the clinical reviewers considered it appropriate [23].

Although the prevalence of inappropriate use reported in the literature varies [2], the figures in our study are comparable to studies in other settings which used the same criteria [14,23]. In other words, up to one quarter of visits to the ER should have been dealt with in the primary health care (PHC) setting. The literature emphasizes overcrowding of services, increased health care costs, and decreased urgent care quality as the main consequences of inappropriate ER use [6-9]. However, it is important to consider that inappropriate use could also have a deleterious impact on those using ER inappropriately. Such patients often receive medications to relieve symptoms but not for the long-term management of their conditions; nor do they receive the results of their exams or follow up visits, i.e. they are not managed in an integrated manner for optimal care [8,13,26].

Older patients had less inappropriate use than younger subjects, consistent with most of the prior literature [1,5,6,16]. This pattern could be due to the higher prevalence of chronic and comorbid diseases among older individuals [27], which more often require immediate attention and complex care (tests and drugs).

As expected, a higher prevalence of inappropriate use was associated with longer stay in the waiting room and longer duration of symptoms [14,16]. This finding suggests that there is quite efficient triage. As found in prior studies [4,14], inappropriate use was higher during the morning shift. Afilalo et al, for example, found that non-urgent patients were less likely to go to the ER between 4:00 PM and 8:00 AM [28].

The study showed that the factors associated with an inappropriate use of the ER differed between two age groups. Among younger individuals, women used the ER more inappropriately than men, in agreement with several studies [6,14]. This result could be related to women's social situation, such as having more free or flexible time, as well as more often being non-workers or unemployed compared to men [6]. Moreover, the women consult more at all levels of care and frequently have facilitated access to health services in general [20].

Within age strata, poor expectations about PHC access (such as lacking other places to go, or having PHC doctors who refuse to see patients without a prior appointment, or a PHC outlet that was open for shorter periods during the day) were associated with inappropriate ER use, which is in agreement with other studies [10,29]. The lack of association between inappropriate ER use and waiting longer for tests, or lacking access to drugs suggests that access to PHC – as opposed to characteristics related to PHC quality – exert a greater impact on inappropriate use of the ER.

Associations between appropriate ER use and having a regular doctor, a defined place for consultation, as well as access to health insurance, have each been described in the literature [9,13,18,20]. However, we did not find these associations, in common with other authors [5]. Just a few subjects with a regular doctor were referred to the ER by a physician, which may indicate difficulty in accessing them. Consistent with the report by Bianco et al [16], our study showed that self or relative-referred patients were more likely to use ER inappropriately.

Some of the variables concerning PHC utilization and access relied on recall over the past 12 months, which could have resulted in recall bias. However, Reijneveld [30] showed good to excellent agreement between retrospective self-report and registered utilization of health care with the same recall period.

Among older patients, level of education had a direct borderline association with inappropriate ER use, indicating disparity in public heath care access. This finding is in agreement with the prior literature [5,7]. Variables related to PHC were not associated with inappropriate use; however, the statistical power for some associations was low. Absence of chronic disease and lack of social support were associated with inappropriate ER use. Most chronic patients ended up having tests performed or being medicated when they attended the ER. However, some of their visits to ER could probably be avoided if the PHC were more easily accessible [27].

The association between lack of social support and inappropriate use is consistent with a review article which reported that older patients lacking social support more often sought care in the ER [17]. Coleman et al, studying patients 60 years or older, found that those who received visits emphasizing self-management of chronic illness, peer support, regular contact with the primary care team, and participation of their spouses and caregivers, tended to end up with fewer ER visits [27]. Despite the scant literature on this subject, the association is plausible and is in agreement with more general studies showing higher access to health services, including PHC services and more appropriate usage among elderly with high social support [31].

## Conclusion

Our study contributes to the understanding of inappropriate use of ER, using a standardized protocol to measure the outcome and suggests that factors associated with inappropriate use varies according to age. Our findings are likely to be generalizable to other Brazilian cities and may be useful for understanding the problem in other countries with a public health system.

There is good evidence of the association between PHC access and inappropriate use of ER in the younger group and among the older group social support might be a marker for the potential benefits of PHC access [31]. Thus, efforts should be made to redirect inappropriate ER demand by expanding access to PHC. This does not mean just expanding services, extending hours of service, or increasing the supply of health professionals [10]. The PHC admission process should be improved, implementing a system of triage that determines the proper place to provide patient care and that addresses the health needs of those who can be attended at the PHC giving them timely advice and treatment. For older patients it is also important to mobilize social support. The relationships between different levels of care need to be enhanced; specifically referring patients back from the ER to their sources of primary care. A sub-group of patients routinely consult at the ER, and this group should be targeted for integration within the PHC system.

Education efforts are also crucial and should focus on how to use health services appropriately, as well as explain to the public about the type of care provided in the ER and the risks and disadvantages of using these services as the primary source of care.

Future studies should take into account the heterogeneous determinants of inappropriate ER use among different age groups. Qualitative approaches would be helpful in furthering our understanding of patient motivations to use the ER inappropriately.

## Competing interests

The author(s) declare that they have no competing interests.

## Authors' contributions

MLVC and AGF designed the project, supervised the field work, performed the data analyses and wrote the paper.

IK contributed to planning the analyses and writing the paper.

## Pre-publication history

The pre-publication history for this paper can be accessed here:


